# Nonclinical prescriptions gave me light of hope: perspectives from people with lived experiences

**DOI:** 10.24095/hpcdp.44.6.08

**Published:** 2024-06

**Authors:** Myrna Norman

**Affiliations:** 1 Canadian Institute for Social Prescribing at the Canadian Red Cross, Mississauga, Ontario, Canada

**Keywords:** social prescribing, self-resilience, hope, community support, dementia

**Highlights**

- A diagnosis of dementia led me to wallow in self-pity and fall into a deep chasm of depression.
- My diagnosis meant I face various challenges with day-to-day activities, including difficulties with word finding, lack of focus to read; I cannot follow or remember recipes.
- The biggest light bulb moment was when I discovered hope.
- Participating in and contributing to supports in my community through social prescribing was key to my health and to lengthening my journey.
- I am living my best life, with hope, and everyone can too. We just need a little help from the doctors and our communities.

Dear Editors,

I was diagnosed with frontal temporal dementia (FTD) in 2008. It was such a frightening diagnosis. My doctor said 5 to 8 years was when my “best-before date” was up. I wallowed in self-pity and just falling into a deep chasm of depression. Then came more doctors and more diagnoses: Lewy body dementia; the doctor revoking my driver’s license; then Alzheimer’s; vascular dementia after my stroke; and, most recently, mild cognitive impairment.

My diagnosis meant I face various challenges with day-to-day activities, including difficulties with word finding, lack of focus to read, inability to follow or remember recipes. I have flooded our kitchen floor twice, which required installing new flooring and more. Short-term memory loss was aggravating.

But I did not want to give up. The biggest lightbulb moment was when I discovered *hope*. After almost two years of struggling to come to terms with my diagnosis, I finally discovered that participating in and contributing to supports in my community was key to my health and to lengthening my journey. 

No doctor ever said to me, “Be happy,” or “Be hopeful,” or “Live your best life.” But that is exactly what I needed. I believe that I would have been able to find my way out of self-pity and depression much sooner if my doctors had given me the tools and opportunity and I didn’t have to navigate it on my own. And I am aware of many persons diagnosed with FTD who need the extra push now.

That is why I am now an active champion for social prescribing, and I am so delighted for the attention and focus that this is receiving now. We can all use a helping hand to find a purpose and a reason to do better every day. I am living my best life, with hope, and everyone can too. We just need a little help from the doctors and our communities.

Yours sincerely,

Myrna Norman

